# Trends in incidence and costs of injuries to the shoulder, arm and wrist in The Netherlands between 1986 and 2008

**DOI:** 10.1186/1471-2458-13-531

**Published:** 2013-06-01

**Authors:** Suzanne Polinder, Gijs IT Iordens, Martien JM Panneman, Denise Eygendaal, Peter Patka, Dennis Den Hartog, Esther MM Van Lieshout

**Affiliations:** 1 Department of Public Health, Erasmus MC, University Medical Center Rotterdam, P.O. Box 2040, 3000 CA Rotterdam, The Netherlands; 2 Department of Surgery-Traumatology, Erasmus MC, University Medical Center Rotterdam, P.O. Box 2040, 3000 CA Rotterdam, The Netherlands; 3 Consumer & Safety Institute, P.O. Box 75169, 1070 AD Amsterdam, The Netherlands; 4 Department of Orthopaedic Surgery, Upper Limb Unit, Amphia Hospital, P.O. Box 90158, 4800 RK Breda, The Netherlands; 5 Department of Emergency Medicine, Erasmus MC, University Medical Center Rotterdam, P.O. Box 2040, 3000 CA Rotterdam, The Netherlands

**Keywords:** Elderly, Fracture, Health care cost, Incidence, Registry, Upper extremity

## Abstract

**Background:**

Upper extremity injuries account for a large proportion of attendances to the Emergency Department. The aim of this study was to assess population-based trends in the incidence of upper extremity injuries in the Dutch population between 1986 and 2008, and to give a detailed overview of the associated health care costs.

**Methods:**

Age-standardized incidence rates of upper extremity injuries were calculated for each year between 1986 and 2008. The average number of people in each of the 5-year age classes for each year of the study was calculated and used as the standard (reference) population. Injury cases were extracted from the National Injury Surveillance System (non-hospitalized patients) and the National Medical Registration (hospitalized patients). An incidence-based cost model was applied in order to estimate associated direct health care costs in 2007.

**Results:**

The overall age-adjusted incidence of upper extremity injuries increased from 970 to 1,098 per 100,000 persons (13%). The highest incidence was seen in young persons and elderly women. Total annual costs for all injuries were 290 million euro, of which 190 million euro were paid for injuries sustained by women. Wrist fractures were the most expensive injuries (83 million euro) due to high incidence, whereas upper arm fractures were the most expensive injuries per case (4,440 euro). Major cost peaks were observed for fractures in elderly women due to high incidence and costs per patient.

**Conclusions:**

The overall incidence of upper extremity injury in the Netherlands increased by 13% in the period 1986–2008. Females with upper extremity fractures and especially elderly women with wrist fractures accounted for a substantial share of total costs.

## Background

Upper extremity injuries account for a substantial proportion of all injury patients visiting the Emergency Departments (EDs). Besides the impact of upper extremity injuries on health and daily life, they impose an economic burden on the community.

The upper extremity consists of the shoulder (*i.e.*, clavicle and scapula), upper arm (i.e., proximal humerus and humeral shaft), elbow (*i.e.*, distal humerus, proximal radius and ulna), forearm (*i.e.*, ulna and radius), wrist (*i.e.*, distal radius and ulna, carpal bones), and hand (*i.e.*, metacarpal bones and the phalanges). Injuries seen in the upper extremity include fractures, dislocations, sprains, contusions, wounds, and superficial lesions.

Population-based knowledge on the economic impact of upper extremity injuries is essential for the allocation of health care services, optimization of preventive measures and research purposes; it also provides a forecast for the future. Most epidemiologic studies on upper extremity injuries primarily focused on one distinct subgroup such as a separate type of injury, anatomical region, or age group [1-15]. In most studies, data from single hospitals or regional data were used [2,3,8,11-13,15-17]. Few publications used a national injury database [1,4,5,7,9,18,19]. Data regarding the associated health care costs are generally lacking. Some studies report direct costs of upper extremity injuries, mostly fractures [2,19-23]. No papers reported on both incidence trends and costs of all injuries to the upper extremity.

Due to budgetary restraints and increasing costs for health care services, economic analyses are becoming more important. The aim of this study was to examine recent long-term population-based trends in the incidence of upper extremity injuries in the Dutch population between 1986 and 2008 and to give a detailed overview of the associated health care costs in 2007.

## Methods

### Data sources

For this retrospective study data were collected for all upper extremity injuries in The Netherlands in the period 1986–2008. Upper extremity injuries were defined using the International Classification of Diseases, ninth revision (ICD-9-CM). All codes in Chapter 17 (Injuries and Poisoning, codes 800–999) related to fractures (810–819), dislocation (830–839), sprains and strains (840–848), open wounds (880–887), superficial injuries (910–919), and contusion (920–924) at the shoulder, arm and wrist area were included. An overview of the ICD-9-CM codes is shown in Table 1. For this study, the upper extremity was separated into shoulder, arm, and wrist. The shoulder region included the clavicle and scapula. The arm region included the upper arm, the elbow, and the forearm. The wrist region included the distal radius, the distal ulna, and the carpal bones.

**Table 1:** Injuries to the shoulder, arm, and wrist as encoded in the ICD-9-CM

**Type of injury**	**ICD-9-CM codes**
**Shoulder**	
Fracture clavicle/shoulder	810, 811
Dislocation shoulder/AC-joint	831
Open wound clavicle/shoulder	880.00, 880.01
Superficial injury/contusion clavicle/shoulder	912, 923.00, 923.01
**Arm**	
Fracture upper arm	812.0, 812.1, 812.2, 812.3
Fracture elbow	812.4, 812.5, 813.0, 813.1
Dislocation elbow	832
Fracture forearm	813.2, 813.3, 813.45, 813.8, 813.9
Open wound arm	881.00, 881.01
Superficial injury/contusion arm	923.1
**Wrist**	
Fracture wrist	813.40, 813.41, 813.42, 813.44, 813.51, 813.52, 813.54, 814
Sprained/dislocated wrist	833, 842
Open wound wrist	881.02, 882
Superficial injury/contusion wrist	914, 923.2

Injury cases were extracted from the National Injury Surveillance System (LIS) [24] and the National Medical Registration (LMR) [25], to include non-hospitalized and hospitalized patients, respectively. The LIS is based upon 13 geographically distributed Emergency Departments (EDs) in the Netherlands, resulting in a representative 12% sample of injury-related ED visits. The adherence population of the participating hospitals in this study is representative for the Dutch population in age and gender structure [24]. The LMR collects data from all Dutch hospitals regarding hospital admissions, admission diagnosis, length of hospital stay, gender, age, and trauma mechanism. With a missing value rate of less than 5% (except 12% for 2007), the LMR data have almost complete national coverage, and were extrapolated to full national coverage [25].

### Calculation of incidence rates and trends

The age-specific incidence rates were calculated in 5-year age groups. For each age group the absolute number of upper extremity injuries was registered in the LIS database. Because the absolute number was obtained from a sample, the figures were weighted in order to create national estimates. An extrapolation factor was estimated by comparing the number of admitted injury patients in the LIS database with the total number of admitted injury patients as recorded in the LMR. The age- and sex-specific incidence rates per 100,000 person years were calculated based upon the Dutch mid-year standard population. The mid-year population sizes for all age-groups were obtained from Statistics Netherlands [26]. “Direct standardization” was used in order to calculate age-adjusted incidence rates [27]. The average number of people in each of the 5-year age classes for each year of the study (1986–2010) was calculated. This number was used as the standard (reference) population, as described previously [28-30]. The overall growth in the number of hospital admissions was calculated for 2008 in percents relative to the year 1986.

### Calculation of costs

The incidence-based Dutch Burden of Injury Model was used in order to measure and describe the health care costs resulting from injuries occurring during a specified period [27]. For each individual injury group patient numbers, health care consumption, and related costs were calculated using the LIS database, the National Hospital Discharge Registry, and a patient follow-up survey conducted in 2007 [31,32]. In this model, the age- and injury-specific costs are based upon the estimated health care supplied to the individual patients. Health care costs of injuries were calculated by multiplication of the incidence, health care volumes (*e.g.*, length of stay in hospital or institution, the number of outpatient visits, General Practitioner visits, home care hours, and physical therapy treatments), and unit costs (*e.g.*, costs per day in hospital). All unit costs were estimated according to national guidelines for health care costing [33]. All costs in this study were calculated over the year 2007. Costs are calculated every five years; the 2007 data were the most recent data available. Despite the 12% of missing data entries for 2007, detailed cost information was available for all patients in the database.

## Results

### Incidence

**Figure 1:**
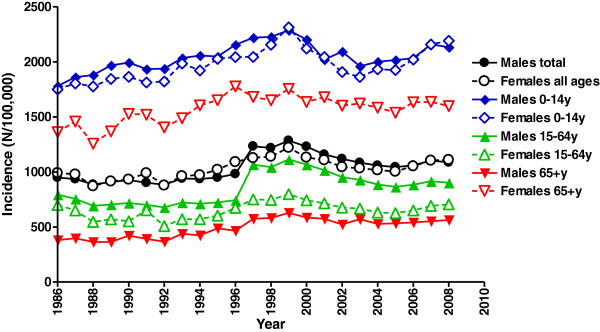
**Age-adjusted incidence (per 100,000 person-years) of upper extremity injuries in the period 1996–2008.** Data are shown for males and females separately.

Between 1986 and 2008, a total number of 3,711,600 patients (1,844,300 males and 1,867,300 females) visited an ED with an upper extremity injury, comprising 42% of the total injury-related ED visits in The Netherlands. The overall (*i.e.,* males and females combined) age-adjusted incidence of upper extremity injuries increased by 13%, from 970 in 1986 to 1,098 in 2008, with a peak in 1999 of 1,250 per 100,000 persons (Figure 1). Since 2005, the incidence increased again, especially in children.

**Figure 2:**
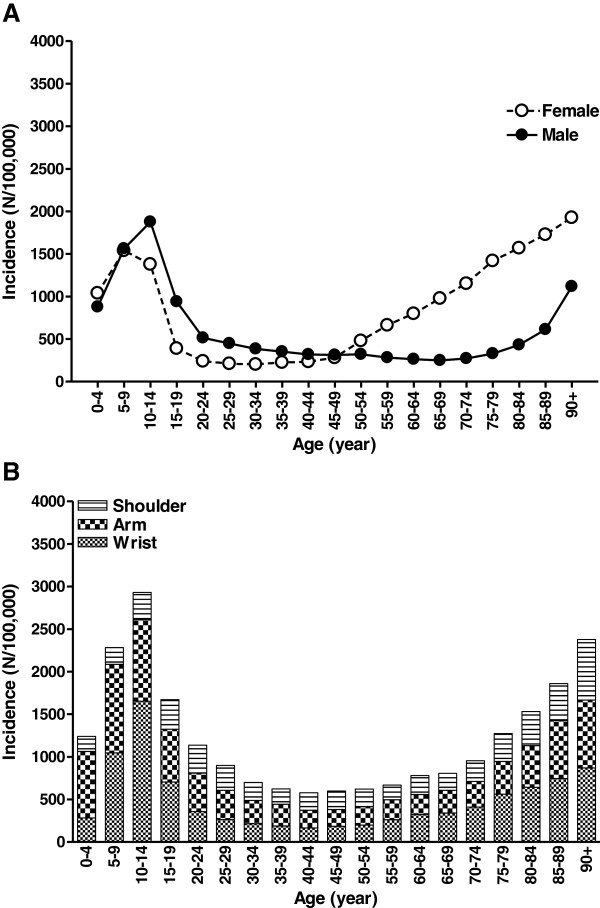
**Age-adjusted incidence (per 100,000 person-years) of upper extremity injuries in the period 1996–2008.** Data are shown by age and gender **(A)** or anatomic location **(B)**.

Injuries to the upper extremity appeared to be age- and gender-related. Women were more likely to sustain an injury to the upper extremity. Over the past two decades, a mean incidence rate for women of 1,042 per 100,000 person-years was seen, compared with 987 per 100,000 for men (Figure 1). Both boys and girls in the age of 5–14 years had a relatively high incidence of upper extremity injuries, especially of the wrist and arm (Figure 2). From the age of 45 onwards, the incidence rate of upper extremity injuries in females increased. In older males, this peak was visible from the age of 80 years onwards.

**Figure 3:**
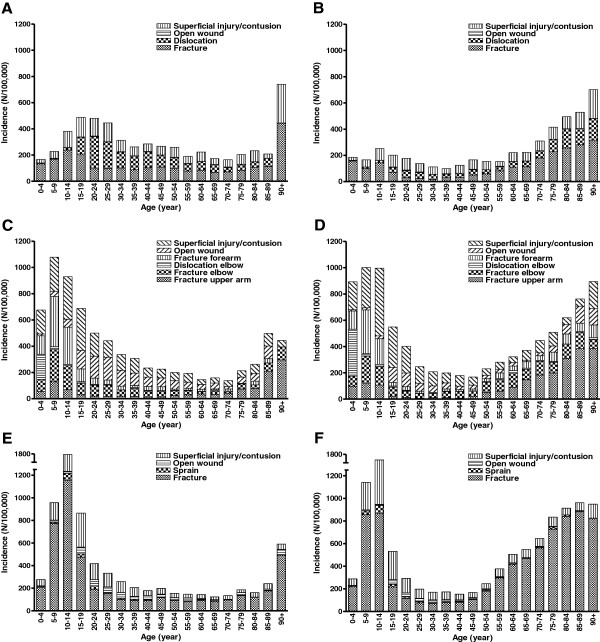
**Age-adjusted incidence (per 100,000 person-years) of the shoulder (A-B), arm (C-D) and wrist (E-F) by age.** Data for 2007 are shown. Data are shown for males **(A, C, E)** and females **(B, D, F)** separately.

The relatively high incidence of upper extremity injuries among boys (aged 10 – 14 year) was mainly attributable to wrist fractures; 1,157 per 100,000 person-years (Figure 3); dislocations and fractures of the shoulder/clavicle were also abundant. Most upper extremity injuries in older women resulted in a fracture, mainly in the wrist and to a lesser extent also in the upper arm (Figures 2B and 3). Superficial injuries/contusions were the most abundant injury in the arm region (32% in males, 33% in females), followed by fractures of the forearm (21% and 20%). Fracture injuries were mainly observed injury in the wrist and shoulder areas and were seen in 61% and 41% of the injuries to the wrist and shoulder, respectively. Wrist fractures occurred more frequently in females than in males (290 versus 206 per 100,000). During the study period, the incidence of wrist fractures increased by 24% in males and by 10% in females.

### Costs

The total health care costs of upper extremity injuries in The Netherlands were €290 million a year, of which 190 million euro were paid for injuries sustained by women (66%; Table 2). Mean costs per patient were €1,150 for males and €2,180 for females. The total health care costs varied substantially between the different injury subtypes. Overall, fractures comprised 53% of all upper extremity injuries but accounted for 76% of the total costs.

**Table 2:** Total cost and cost per case of all injuries of the upper extremity

	**Overall**			**Males**			**Females**		
	**N cases**	**Total cost**	**Cost per**	**N cases**	**Total cost**	**Cost per**	**N cases**	**Total cost**	**Cost per**
		**(€)**	**case (€)**		**(€)**	**case (€)**		**(€)**	**case (€)**
**Shoulder**	**38,776**	**70,418,210**	**1,820**	**23,324**	**29,811,080**	**1,280**	**15,452**	**40,607,130**	**2,630**
Fracture	16,647	42,422,680	2,550	9,646	16,434,470	1,700	7,001	25,988,220	3,710
Dislocation	10,167	17,499,320	1,720	6,938	8,643,750	1,250	3,229	8,855,570	2,740
Open wound	281	282,830	1,010	215	222,210	1,030	66	60,620	920
Superficial injury/contusion	11,681	10,213,380	870	6,524	4,510,650	690	5,156	5,702,720	1,110
**Arm**	**67,674**	**121,060,450**	**1,790**	**32,652**	**41,426,120**	**1,270**	**35,022**	**79,634,330**	**2,270**
Fracture upper arm	9,038	40,143,150	4,440	3,088	8,789,630	2,850	5,949	31,353,520	5,270
Fracture elbow	11,809	28,225,280	2,390	5,163	8,517,370	1,650	6,646	19,707,910	2,970
Dislocation elbow	3,625	4,174,760	1,150	1,482	1,478,510	1,000	2,143	2,696,250	1,260
Fracture forearm	11,266	25,894,070	2,300	5,992	12,034,840	2,010	5,274	13,859,220	2,630
Open wound	9,542	8,277,780	870	6,327	4,893,220	770	3,215	3,384,570	1,050
Superficial injury/contusion	22,395	14,345,420	640	10,600	5,712,550	540	11,795	8,632,870	730
**Wrist**	**67,540**	**98,791,390**	**1,460**	**30,630**	**28,584,230**	**930**	**36,910**	**70,207,160**	**1,900**
Fracture	44,019	83,208,720	1,890	18,819	21,657,900	1,150	25,200	61,550,820	2,440
Sprain	2,478	1,946,670	790	1,172	852,820	730	1,306	1,093,860	840
Open wound	3,305	3,127,140	950	2,288	1,955,250	850	1,017	1,171,890	1,150
Superficial injury/contusion	17,737	10,508,860	590	8,350	4,118,270	490	9,387	6,390,590	680
**Total**	**173,989**	**290,270,050**	**1,670**	**86,605**	**99,821,440**	**1,150**	**87,384**	**190,448,620**	**2,180**

Women with wrist fractures accounted for 21% of total costs of upper extremity injuries. The total health care costs for wrist fractures were €83 million making them the most expensive injuries. This seemed mainly attributable to the high incidence (Table 2). Upper arm and shoulder fractures represented 5% and 10% of all injuries, respectively. However, with total costs of over €40 million each, they were the second and third most expensive injuries. Fractures of the wrist, shoulder, and upper arm accounted for almost 60% of total costs. With average costs of €4,440 per case, upper arm fractures represented the most expensive injury per case in both men and women.

A substantial difference in costs between males and females was noted. For almost all injury groups total costs were higher for females, except for open wounds (Table 2). Costs were generally higher due to higher incidence rates and higher mean costs. An average upper extremity injury in women aged 65 years or older was with €4,310 per case approximately €1,400 more expensive than the same injury in men.

**Figure 4:**
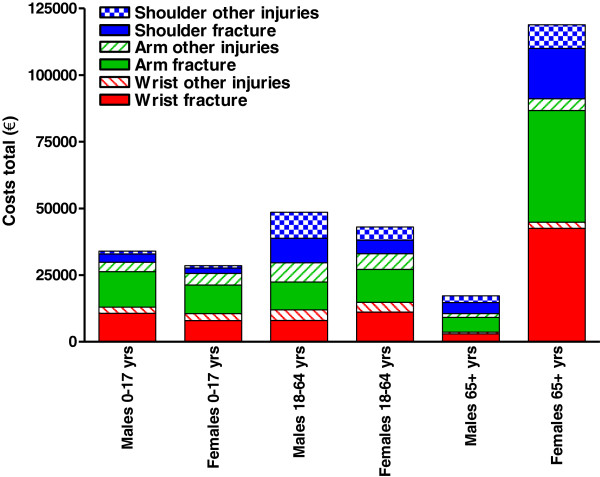
**Total costs related to injuries of the shoulder, arm and wrist.** Data for 2007 are shown, subdivided into three age groups for males and females.

Figure 4 shows the total cost per type of injury by gender for three age groups. Although the total costs for the male population under the age of 65 was slightly higher than for their female peers, the total costs for females aged 65 years or older was almost seven times higher than the corresponding age group in males, mainly due to fractures and dislocations.

## Discussion

Upper extremity injuries accounted for 42% of all injury-related visits to the Emergency Departments (EDs). In the past 25 years the overall incidence of upper extremity injuries in the Netherlands increased by 13%. Throughout the years, the incidence was age and gender related. The increase in incidence of upper extremity injuries is most evident in patients aged 60 years and above. Fractures are the most expensive type of injury, especially in women.

Our data demonstrate an evident influence of age and gender on the incidence of certain upper extremity injuries. The 10–14 year old boys group is prone to wrist fractures, as shown before [34,35]. During this age, an increased calcium demand combined with maximal skeletal growth and an increased physical activity leads to more fractures [35]. Young males have a higher upper extremity injury incidence than females of the same age, which seems in line with previous findings that young males experience more road traffic incidents and sports trauma [3,19]. Women suffer significantly more fractures when aged 65 years and over, which seems attributable to the increasing occurrence of postmenopausal osteoporosis in elderly women [6,9,10,36]. An equal rise in humeral fractures in females of this age-group supports this. In addition, the higher rate of falls may also explain the rise in fractures in the elderly [29,30].

Several studies describe incidence rates on injuries that were also included in the current study. Since these used another reference population form the standardization, absolute numbers may differ. However, trends remain indicative. Lofthus *et al.* reported that incidence rates on wrist fractures in females aged 50 and over range from 554 to 1,098 per 100,000 [16]. This seems slightly higher than the incidence found in our study (average 489, range 430–621 per 100,000), but this may be due to differences in the reference population. In literature, dislocation of the glenohumeral joint ranged from 11.2-27.0 per 100,000 person years [1,37,38]. This is lower than the incidence (51.2 per 100,000) found in our study, which also contained dislocation of the acromioclavicular joint. In accordance with our data, all studies displayed a higher incidence of shoulder dislocations in men than in women [1,37,38].

Even though the age-adjusted incidence rates for men and women were similar, the total costs of upper extremity injuries for females almost doubled those of males. This huge difference is for a considerable part attributable to the higher costs per case in females and the female preponderance in the older Dutch population (Statistics Netherlands) [26]. Over 75% of total costs were attributable to fractures, making them the most expensive injuries. The majority of the costs for fractures were accounted for by women (69%). Fractures were expected to have the highest costs of all injuries, due to possible hospital admissions, surgical intervention, plaster treatment, X-rays, longer rehabilitation, and physical therapy. An explanation for the extensive costs of fractures in the elderly females could be that osteoporotic bones of postmenopausal women fracture more severely [37]. Such fractures may require more radiological evaluation and more extensive or expensive surgical interventions. Also, new surgical techniques may have lowered the threshold for surgical interventions. In addition, surgery performed in osteoporotic bone has a higher failure rate which may result in an increased rate of revision surgeries [39]. A final explanation for the higher costs of fracture care in the elderly women could be that they outlive their partners, which may increase the chance of extended nursing home admission or home care.

To the best of our knowledge, this is the first population-based study to show trends in incidence and cost of fourteen different injuries of the upper extremity at a national level. A few other studies presented cost information of upper extremity injuries, of which most concern high-risk groups [2,21] or economic evaluation studies of treatment interventions [40,41]. Only Meerding *et al.* calculated costs of fractures of the wrist, the clavicle/shoulder, and the upper arm in the Netherlands [19]. After applying a correction for inflation, Meerding *et al.* reported €1,080 for wrist fractures, €1,130 for clavicle/shoulder fractures, and €3,200 for upper arm fractures, as opposed to €1,890, €2,550 and €4,440, respectively, in the current study. The higher costs as observed in the current study may be attributable, at least partly, to a higher number of patients receiving operative treatment for fractures. Higher current costs for (new or improved) implants can also not be ruled out. Finally, recent improvement in the data sources on home and nursing care and on operative interventions may have resulted in a more accurate, most likely higher, estimate of costs in our study.

The main strength of our study is that we used up-to-date population-based data over a longer, continuous time-period. The use of data from a representative national sample of outpatients using data from a national registry is a more reliable representation of the health care problem than extrapolating data from one clinical trial or one hospital only [24]. Although the registrations in the LIS-database only cover 12% of the Dutch population, international validation studies have shown that the mathematical model that was applied for the calculation of the overall Dutch data has a high level of completeness and validity. Meerding *et al.* showed that there was a close agreement between de cases recorded in the LIS and the hospital’s discharge system [24]. Lyons *et al.* reported that there was a particularly good agreement between the extrapolated data from the LIS and the actual incidences of hospital admissions for injuries [42]. Another strength of our study is that it presents comprehensive estimates of health care costs, including all relevant health care sectors (*i.e.*, hospital inpatient care, medical procedures, rehabilitation clinics, and nursing homes). The model uses data from the LIS, the National Hospital Discharge Registry, and a patient follow-up survey conducted in 2007. Unfortunately, when performing the follow-up survey, it was not known that 2007 was a year with relatively more missing data. However, due to the very large sample of the survey and the use of a uniform coding method, it was possible to compare the healthcare use and related healthcare costs of all types of upper extremity injuries [31].

A limitation of the cost model is that indirect health care costs, such as absenteeism and work disability were not taken into account. This could be a suggestion for future research. Furthermore, there may be some statistical uncertainty due to underreporting of combined injuries. For example, patients with wounds concomitant with a fracture will be reported as fractures, not as wounds. Moreover, only patients who visited the ED were recorded in the LIS and LMR databases. Therefore patients who visited their general practitioner were not included.

## Conclusions

There has been a 13% rise in incidence of upper extremity injuries in the Netherlands over the past two decades. These injuries constitute a substantial part of all injury-related ED visits and impose a burden on health care costs. The incidence of upper extremity injuries seems strongly age and gender related. Fractures are the most common injuries and they impose the greatest burden on health care costs, especially in women. Current treatment programs of especially frequently occurring injuries and injuries associated with high costs need to be evaluated in order to assess if health care cost reduction is feasible.

## Abbreviations

ED: Emergency department; ICD-9-CM: International classification of diseases 9th revision, clinical modification; LIS: National injury surveillance system; LMR: National medical registration.

## Competing interests

The authors declare that they have no competing interests.

## Authors’ contributions

SP participated in the design of the study and the statistical analysis and drafting of the manuscript. GITI participated in the design of the study, and assisted in the data analysis and data interpretation, and drafting of the manuscript. MJMP participated in the design of the study, data collection and critical revision of the manuscript. DE participated in the design of the study, data interpretation and critical revision of the manuscript. PP participated in the design of the study, data interpretation and critical revision of the manuscript. DDH participated in the design of the study, data interpretation and critical revision of the manuscript. EMMVL supervised, and participated in the design of the study, data collection, analysis and interpretation, and drafting and critical revision of the manuscript. All authors have read and approved the final manuscript.

## Pre-publication history

The pre-publication history for this paper can be accessed here:

http://www.biomedcentral.com/1471-2458/13/531/prepub
